# A new species of *Hibiscadelphus* Rock (Malvaceae, Hibisceae) from Maui, Hawaiian Islands

**DOI:** 10.3897/phytokeys.39.7371

**Published:** 2014-07-25

**Authors:** Hank L. Oppenheimer, Keahi M. Bustamente, Steven P. Perlman

**Affiliations:** 1 Plant Extinction Prevention Program, Pacific Cooperative Studies Unit, University of Hawai`i/ Manoa, Dept. of Botany, P.O. Box 909, Makawao, HI 96768, USA; 2 National Tropical Botanical Garden, 3530 Papalina Rd., Kalaheo, HI 96741, USA

**Keywords:** Malvaceae, *Hibiscadelphus*, Hawaiian Islands, Maui, conservation, IUCN Red List

## Abstract

*Hibiscadelphus stellatus* H. Oppenheimer, Bustamente, & Perlman, **sp. nov.**, a new, narrowly endemic species from West Maui, Hawaiian Islands is described, illustrated and its affinities and conservation status are discussed. It is currently known from three populations totaling 99 plants in Kaua`ula valley on leeward western Maui. It differs from *H. wilderianus*, its nearest congener, in its denser white or tan stellate pubescence on most parts; larger externally purple colored corollas that are 5–6.5 cm long; linear-subulate to lanceolate, acute to acuminate involucral bracts; globose-cuboid to ovoid capsules; and endocarp with scattered hairs.

## Introduction

Joseph Rock described the endemic Hawaiian genus *Hibiscadelphus* Rock in 1911 based on *Hibiscadelphus giffardianus* Rock (Radlkoffer and Rock 1911). The genus is extremely rare, with seven previously described species from the main Hawaiian Islands, four of which are now extinct, two only persisting in cultivation (including restoration plantings), and a single species remaining in its natural habitat. The genus belongs to the tribe Hibisceae (Malvaceae), and it appears to form a distinct monophyletic group based on its curved and narrowly zygomorphic corollas forming a tubular structure with the petals unequal in length (the lower two shorter than the upper three). In contrast, in *Hibiscus* the corollas are actinomorphic with spreading petals of equal length (Lorence and Wagner 1995). In most species of *Hibiscus* the calyx is not circumscissile in fruit but persists, splitting along one side.

In addition to establishing the genus, Rock described three species: *Hibiscadelphus giffardianus* Rock from Mauna Kea, *Hibiscadelphus hualalaiensis* Rock from Hualalai, both on Hawai`i Island, and *Hibiscadelphus wilderianus* Rock from Auwahi on the island of Maui (Rock 1913). After Rock’s initial treatment, Forbes (1920) described a fourth species (*Hibiscadelphus bombycinus* C.N. Forbes) based on a specimen collected in the mid 1800’s by Hillebrand and Lydgate at Kawaihae in the Kohala Mountains of Hawai`i Island. Over the next 75 years three additional species were subsequently discovered and described: *Hibiscadelphus distans* L.E. Bishop & D. R. Herbst on Kaua`i (Bishop and Herbst 1973); *Hibiscadelphus crucibracteatus* Hobdy on Lana`i (Hobdy 1984), and *Hibiscadelphus woodii* Lorence & W.L. Wagner on Kaua`i (Lorence and Wagner 1995). The last authors published a key to the seven taxa known at that time. Presently, six species are extinct in the wild, but two of these persist in cultivation (including restoration outplantings), and two others, including this new species, occur as natural populations (Table 1). The eight described species are all mostly single volcano endemics. The two Kaua`i species are separated by a distance of 8 km. *Hibiscadelphus woodii* was known from Kalalau Valley on the islands northern coast and *Hibiscadelphus distans* is known from Koaie Stream in Waimea Canyon, whose outlet is along the southern shore

**Table 1.** Current status of *Hibiscadelphus*.

	Extinct	Cultivation only	Extant in wild	USFWS status	IUCN status
*Hibiscadelphus bombycinus*	×			Species of Concern	EX
*Hibiscadelphus crucibracteatus*	×			Species of Concern	EX
*Hibiscadelphus distans*			×	Endangered	CR
*Hibiscadelphus giffardianus*		×		Endangered	CR
*Hibiscadelphus hualalaiensis*		×		Endangered	CR
*Hibiscadelphus stellatus*			×		EN
*Hibiscadelphus wilderianus*	×			Species of Concern	EX
*Hibiscadelphus woodii*	×			Endangered	CR

During the course of field work on west Maui in 2012 the authors discovered two populations (25 and 51 plants) over 400 m apart of a previously unknown *Hibiscadelphus* species on the steep slopes of Kaua`ula Valley on leeward, western Maui. A year later a third colony was found between the first two locations with 23 plants. *Hibiscadelphus* had not been observed, reported or documented previously on west Maui. Study of the collected specimens and comparison with collections of other known species at the BISH and PTBG herbaria, and images on JSTOR Global Plants revealed they represent an undescribed species.

## Taxonomy

### Hibiscadelphus stellatus

Taxon classificationPlantaeMalvalesMalvaceae

H. Oppenheimer, Bustamente, & Perlman sp. nov.

urn:lsid:ipni.org:names:77140885-1

Figs 1 , 2

#### Note.

Differs from *Hibiscadelphus wilderianus* in its denser pubescence especially on leaves, petioles, peduncles, involucral bracts, and corolla; linear-subulate to lanceolate involucral bracts, with acute to acuminate apices; evenly 5-lobed calyx; wider, densely pubescent, externally purple, internally yellow corolla lobes; and ovate to sub-globose capsules, 2.5–3.5 × 2.2–3.2 cm with scattered long hairs on the endocarp.

#### Type.

**USA. HAWAIIAN ISLANDS:** West Maui, Lahaina District, Kaua`ula Valley, south slope, 841 m, 13 Feb 2014, Oppenheimer, Bustamente & Perlman H21404 (holotype: BISH; isotypes: MO, NY, PTBG, US).

#### Description.

Small trees 3–6 m tall, many branched, trunks to 30 cm dbh, bark smooth, light tan to gray, young branchlets densely white to tan pubescent with 8–12-rayed stellate trichomes 0.3–0.4 mm in diam., surface scurfy-waxy, glabrescent with age; petiole scars prominent, subcircular, 2.5–4 mm in diam. Leaves chartaceous, new growth densely stellate-pubescent, mature leaves with blades broadly-ovate to suborbicular or subreniform in outline, occasionally shallowly 3-lobed, 7.5–16(–18) cm long, (8)9.5–13.5(–18) cm wide, veins prominulous, primary veins 7–9 radiate from base, midvein with 3–4 pairs of secondary veins arising along midrib, light green to occasionally red tinged when fresh, higher order venation prominulous on both surfaces, margins irregularly broadly crenate, base cordate, with a wide to narrow but usually open sinus, apex acute to obtuse or rounded, green when fresh with scattered tan stellate pubescence on both surfaces, densely so along veins and adaxial surface, trichomes 0.2–0.4 mm in diam. with (2–)8–16 rays, abaxial surface with principal vein axils domatiate with dense tufts of tan to white trichomes 0.2–0.3 mm long; petioles 3.5–6cm long, green or sometimes red-tinged, pubescent with dense white to tan stellate trichomes as on branchlets; stipules lanceolate to subulate, 2–3.5 mm long, apex acute, green, sparsely to densely tan or white stellate pubescent, soon caducous. Flowers solitary, axillary, erect to spreading, pedicels 22–30 mm long, green or sometimes red-tinged, densely white to tan stellate pubescent as in petioles, involucral bracts 5–6 (–7), linear-subulate to lanceolate (rarely spathulate), acute to acuminate apically, connate only at base, 9–22 mm long, 1–2 mm wide at base, erect, appressed or spreading perpendicular to the floral axis in anthesis, green, densely tan or white stellate pubescent with trichomes 0.2–0.3 mm in diam. Calyx tubular-saccate, mostly 5-lobed, tube 22–30 mm long, 19–20 mm wide, the lobes triangular, acute to short acuminate 5–10 mm long, 7–8 mm wide, green, surface obscured by dense tan stellate pubescence as in bracts, in mature fruit splitting along one side but persistent. Corolla zygomorphic, adaxially curved, 5–6.5 cm long, lobed nearly to base, lobes coalescent, 6–6.5 cm long, 3.5–4 cm wide, obovate-spathulate, apex obtuse, tips and outer margins slightly reflexing with age, outer exposed portion purple, purple-green or purple-yellow, inner concealed portion yellow, conspicuously veined, densely covered with gray or tan stellate trichomes especially along veins, internally yellow or purple-tinged distally, purple toward base, corolla usually becoming purplish with age, staminal column and apex of the style exserted for 1.5–2.5 cm; staminal column 8–8.5 cm long, antheriferous in distal 3.5 cm, maroon-purple, antheriferous in distal 3.5 cm, stamens c. 100, anthers reniform-curved, 0.8–1.5 mm long, purple, filaments 6–12 mm long, purple, pollen grains purple turning golden yellow after anther dehiscence; style 8.5–9 cm long, style branches 3–5 mm long, villose, stigmas rounded, c. 1 mm long, yellow, ovary dome-shaped, 8 mm long and wide. Fruit a woody capsule, globose-cuboid to -ovoid, 5-locular, 5-valved, 2.5–3.5 (–4) cm long, 2.2–3.3 cm in diameter, surface yellowish brown, rough densely covered with dense tan stellate hair clusters, appearing tuberculate, mericarps 10, mesocarp well developed, reticulate, endocarp chartaceous, loose, with scattered long hairs, testa brown. Seeds 1–2 per mericarp, reniform, 8–10 mm long, 6–8 mm wide including the dense, lanate yellowish-tan hairs 0.4–1 mm long.

**Figure 1.**
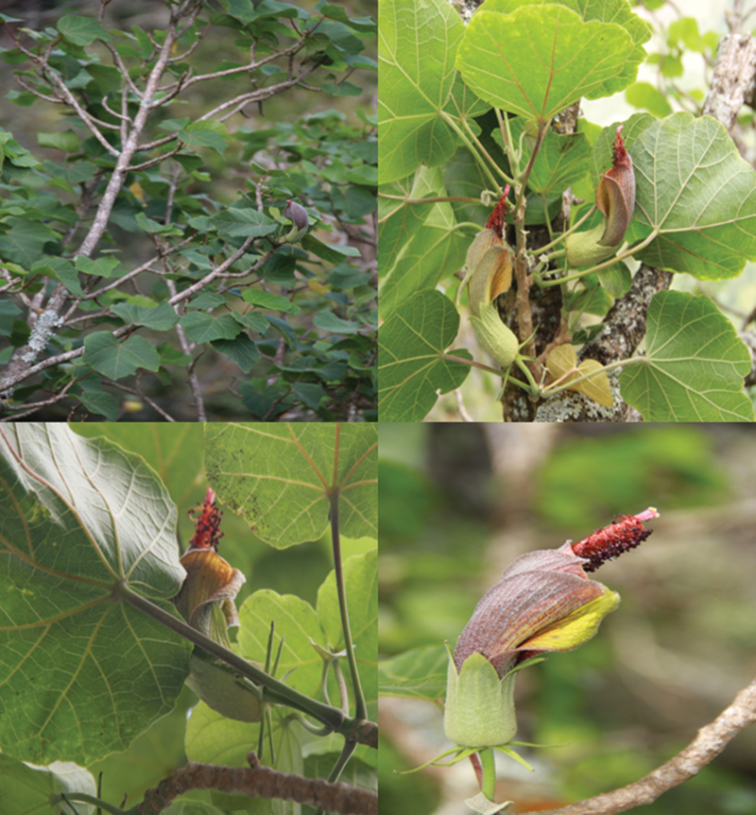
*Hibiscadelphus stellatus*. **A** Habit **B** Flowers and leaves (from the holotype) **C** View of bracts illustrating stellate arrangement **D** Close-up of flower. (from the holotype). All photos by the authors.

**Figure 2.**
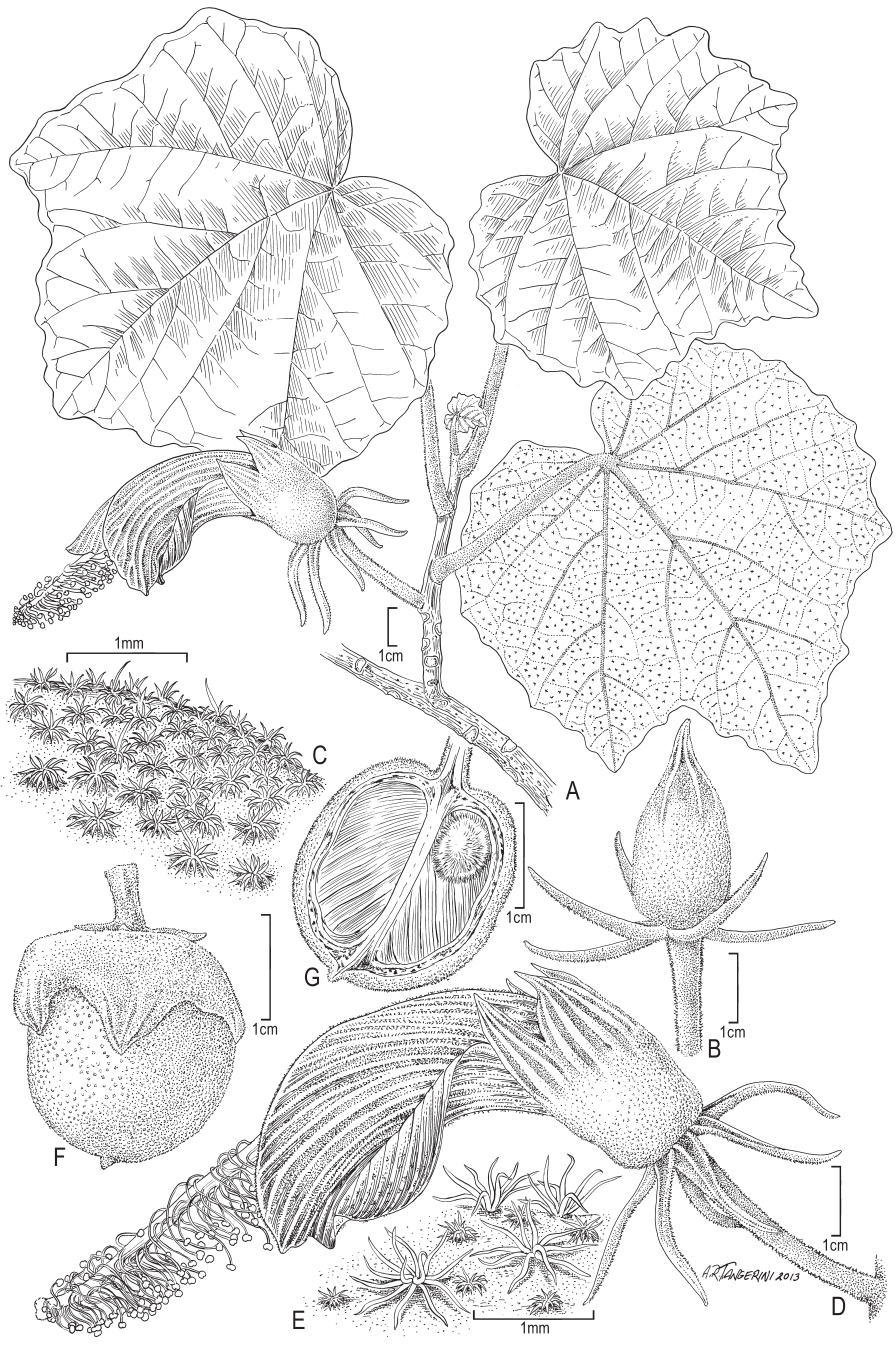
*Hibiscadelphus stellatus* H. Oppenh., Bustamente, & Perlman. **A** Habit **B** Flower bud **C** Surface of calyx showing stellate hairs **D** Flower **E** Surface of corolla showing two sizes of stellate hairs **F** Fruit **G** Longitudinal section of fruit showing seed. Drawn from Oppenheimer et al. H41337 (US) and field photographs by the authors.

#### Distribution.

**Figure 3.**
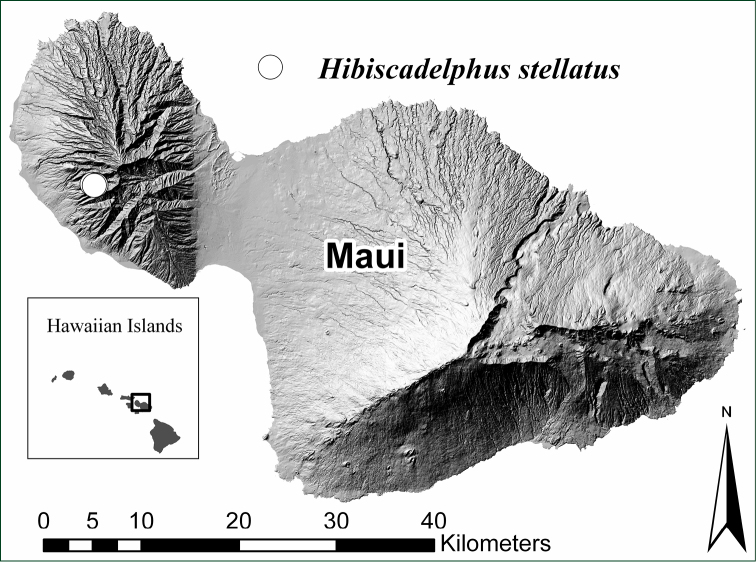
Distribution map showing known locations of *Hibiscadelphus stellatus* on West Maui.

Known only from west Maui, Hawaiian Islands at 20.87°N, 156.62°W (Fig. 3).

#### Habitat and ecology.

*Hibiscadelphus stellatus* occurs on very steep, rocky slopes between 800 and 900 m elevation. These sites have a windward aspect and are situated mid-slope between the upper rim of a deep valley and a perennial stream below. Soils at these sites are of typical volcanic, basalt origin, from the Wailuku Series of original shield building flows. The vegetation where *Hibiscadelphus stellatus* grows forms a mosaic of trees and shrublands with an open canopy, best characterized as Lowland Mesic Forest (Wagner et al. 1999). Rainfall averages from 12 to 1400 mm annually and the substrate is well-drained.

Associated tree species include: *Alectryon macrococcus* Radlk., *Antidesma pulvinatum* Hillebr., *Coprosma foliosa* A. Gray, *Diospyros sandwicensis* (A. DC) Fosberg, *Dodonaea viscosa* Jacq., *Metrosideros polymorpha* Gaudich. var. *glaberrima* (H. Lév.) H. St. John, *Myoporum sandwicense* A. Gray, *Myrsine lanaiensis* Hillebr., *Myrsine lessertiana* A. DC., *Nestegis sandwicensis* (A. Gray) O. Deg, I. Deg. & L.A.S. Johnson, *Pisonia sandwicensis* Hillebr., *Pittosporum confertiflorum* A. Gray, *Pouteria sandwicensis* (A. Gray) Baehni & O. Deg., *Psychotria kaduana* (Cham. & Schltdl.) Fosberg, *Psydrax odorata* (G. Forst.) A.C. Smith & S.P. Darwin, *Santalum ellipticum* Gaudich., *Sophora chrysophylla* (Salisb.) Seem., *Streblus pendulinus* (Endl.) F. Muell., *Zanthoxylum dipetalum* H. Mann, and *Zanthoxylum hawaiiense* Hillebr. Understory species include: *Achyranthes splendens* Mart. ex Moq., *Bidens micrantha* Gaudich., *Charpentiera ovata* Gaudich., *Euphorbia multiformis* Gaudich. ex Hook. & Arn., *Osteomeles anthyllidifolia* (Sm.) Lindl., *Pipturus albidus* (Hook. & Arn.) A. Gray, *Pleomele auwahiensis* H. St. John, *Remya mauiensis* Hillebr., *Urera glabra* (Hook. & Arn.) Wedd., and *Wikstroemia oahuensis* (A. Gray) Rock. Ferns are locally common in the understory and include: *Asplenium nidus* L., *Doodia kunthiana* Gaudich., *Dryopteris sandwicensis* (Hook. & Arn.) C. Chr., *Lepisorus thunbergianus* (Kaulf.) Ching, and *Microlepia strigosa* (Thunb.) C. Presl. Vines are represented by *Alyxia stellata* (J.R. Forst. & G. Forst.) Roem. & Schult., *Ipomoea tuboides* O. Deg. & Ooststr, *Lipochaeta connata* (Gaudich.) DC, *Sicyos pachycarpus* Hook. & Arn., and *Smilax melastomifolia* Sm. Grasses and sedges are sparse and include: *Eragrostis variabilis* (Gaudich.) Steud., *Panicum nephelophilum* Gaudich., *Trisetum inaequale* Whitney, *Carex meyenii* Nees, and *Carex wahuensis* C.A. Mey.

#### Phenology.

*Hibiscadelphus stellatus* has been observed with buds, flowers and immature and mature fruit capsules in February and April. Flowers open mid-day and produce abundant nectar.

#### Etymology.

*Stellatus* – Latin, star shaped, alluding to the stellate pubescence that characterizes the Malvaceae in general, including *Hibiscadelphus*. The name also refers to the “star-shaped” pattern formed by the five involucral bracts, which contrasts with the cruciform pattern formed by the four bracts in *Hibiscadelphus crucibracteatus*. Additionally, *stellatus* acknowledges the beautiful and stellar (outstanding) flowers of this species. The Hawaiian name *hau kuahiwi* has been applied to other species of the genus (Rock 1913). *Hau* (*Hibiscus tiliaceus* L.), a lowland tree; *kuahiwi*–*lit.* mountain or high hill (Pukui and Elbert 1986). Hawaiians recognized the similarities of the taxa while observing that *Hibiscadelphus* grows at higher elevations.

#### Conservation efforts.

The conservation status of *Hibiscadelphus* is precarious at best. Three species (*Hibiscadelphus crucibracteatus*, *Hibiscadelphus giffardianus*, and *Hibiscadelphus wilderianus*) were each only known from a single naturally occurring tree (Hobdy 1984; Rock 1913). However, *Hibiscadelphus giffardianus* survives in cultivation and is planted within the type locality at Kipuka Puaulu in what is now Hawai`i Volcanoes National Park. Hillebrand provided no information on the abundance or scarcity of *Hibiscadelphus bombycinus* when he first collected it but the species is presumed extinct. *Hibiscadelphus crucibracteatus* is presumed extinct in the wild since the single known tree died a few years after its discovery from damage by introduced axis deer (*Axis axis*) despite it being fenced; there is no ex situ material although there were several attempts at propagation (R. Hobdy, pers. comm.). *Hibiscadelphus woodii* was known from four individuals, but evidently has recently gone extinct (Wood 2012). There are no plants in cultivation despite attempts to propagate it. *Hibiscadelphus hualalaiensis* is considered extinct in the wild as of 1992 but is in cultivation. *Hibiscadelphus wilderianus* is also presumed extinct. Although Rock mentioned that Wilder (who discovered the species with Rock, later returning and making several additional collections from the only known tree) had succeeded in raising a single seedling (Rock 1913) no surviving material is known. *Hibiscadelphus distans* is known from two wild populations of approximately 15–20 individuals total on Kaua`i, and over 100 ex situ collections at the McBryde and Limahuli gardens of the National Tropical Botanical Garden (NTBG). With 99 known plants, *Hibiscadelphus stellatus* has the largest known wild populations plus the only known naturally occurring seedlings of any species in the genus.

Seeds were collected from 12 individuals of *Hibiscadelphus stellatus* representing the three known subpopulations. The subpopulations were mapped with GPS and each individual plant numbered and tagged. Cuttings from three plants were also made although these failed to take root. Material is being propagated at the Olinda Rare Plant Facility on Maui, NTBG on Kaua`i and the Lyon Arboretum on O`ahu. The first seeds germinated in conventional propagation approximately 50 days after sowing and under three weeks in tissue culture. As of May 2013 four parent trees from two sites are represented ex situ, with seeds from four additional trees in the third site now in propagation at Olinda and Lyon.

Threats to the existence of *Hibiscadelphus stellatus* include habitat erosion, fire, weeds, drought, probably rats (*Rattus rattus*, *Rattus exulans*) (Baker and Allen 1978) and mice, (*Mus domesticus*), slugs such as *Derocerus* and *Limax* or other invertebrates such as seed weevils (Giffard 1920) and caterpillars (Lorence and Wagner 1995), and potentially feral goats (*Capra hirca*) and/or pigs (*Sus scrofa*). Small populations of feral goats and pigs are encroaching in surrounding areas, although the West Maui Mountains Watershed Partnership is constructing strategic fencing. In 2007 a large wild fire burned within 180 m of the plants; succession of its habitat presently includes non-native fire-adapted grasses that were absent before the fire. Erosion is a natural process but is exacerbated by invasion by weeds and ungulates and the destruction of vegetation by fire. Woody non-native plants are currently low in diversity and number, but are represented by known aggressive, habitat – modifying species such as *Grevillea robusta* A. Cunn. ex R. Br., *Lantana camara* L., *Psidium guajava* L., and *Schinus terebinthifolius* Raddi. Herbaceous understory weeds are similarly low in number of taxa but include serious habitat modifiers such as *Adiantum hispidulum* Sw., *Ageratina adenophora* (Spreng.) R.M. King & H. Rob., *Ageratina riparia* (Regel) R.M. King & H. Rob., *Buddlea asiatica* Lour., *Erigeron karvinskianus* DC., and *Oplismenus hirtellus* (L.) P. Beauv., all of which may hinder establishment of seedlings.

#### Conservation status.

When evaluated using the IUCN Red List criteria (IUCN 2013) *Hibiscadelphus stellatus* falls into the Endangered (EN) category, a designation for taxa facing a very high risk for extinction in the wild. The species merits this designation by meeting the following criteria: B2(a)(biii, v) + D, where the area of occupancy (AOO) is less than 500km² (B2), with severely fragmented or number of locations <5 (a), and a continuing decline observed, estimated, inferred or projected in (biii) quality of habitat and (bv) number of mature individuals; and D: <250 mature individuals. Although there is some reproduction observed, there is not a sufficient population structure that will allow enough immature plants to replace mature individuals as they perish, therefore a decline is almost a certainty under current conditions. The AOO is 2.28 hectares (5.63 acres) much less than the threshold. The habitat is inferred to be in decline due to the effects of introduced taxa such as invasive plants and rats, as well as the effects of introduced rats and diseases on pollinators. Continued monitoring over the next five years will possibly lead to an updated assessment to CR. Furthermore we recommend that the U.S. Fish & Wildlife Service list this new species as Endangered under the Endangered Species Act of 1973 and that the Service prepare and fund a recovery plan.

#### Specimens examined.

**USA.** Hawaiian Islands: Maui: west Maui, Lahaina District, Kaua`ula Valley, south side slope, 807 m, 17 Apr 2012, *Oppenheimer et al. H41214* (BISH), 841 m, 24 Apr 2013, *Oppenheimer et al. H41337* (US), 13 Feb 2014, *Oppenheimer et al. H21403* (BISH), *H21406* (BISH), *H21407* (BISH), 820 m, *Perlman, et al. 23853* (PTBG); Kaua`ula valley, below Helu, 817 m, 17 Apr 2012, *Perlman et al. 22834* (PTBG, 2 sheets), Kaua`ula valley, south slope below Helu summit, 817 m, 18 Apr 2012, *Perlman et al. 22837* (PTBG).

#### Discussion.

This new species clearly belongs to *Hibiscadelphus* based on its flowers that have their corolla lobes coalescent into a curved, tubular zygomorphic structure. *Hibiscadelphus stellatus* differs from its congeners in the following combination of characters: moderate to dense stellate pubescence on all parts; involucral bracts 5 (–7) in number that are linear-subulate to lanceolate, 9–22 mm long, and acute to acuminate apically; 5-lobed calyx with tube 22–25 mm long and lobes 5–8 mm x 7–8 mm; externally purplish-colored corolla 5–6.5 cm long; and globose-cuboid to ovoid capsules with scattered hairs on the endocarp. The species of *Hibiscadelphus* can be separated by the following key.

### Key to the species of *Hibiscadelphus*

**Table:** 

1a	Involucral bracts connate ca. ½ of their length; mesocarp weakly developed and usually adnate to the exocarp; endocarp segments 5	*Hibiscadelphus distans*
1b	Involucral bracts free or slightly connate at base; reticulate mesocarp strongly developed; endocarp segments 10	2
2a	Involucral bracts filiform or obsolete; up to 1.1 mm wide toward base	3
2b	nvolucral bracts linear-subulate to spathulate, 1–7 mm wide toward base	4
3a	Involucral bracts 0.5–2(–3) mm long; corolla greenish yellow externally, fading to purplish internally, 2–5(–5.5) cm long; Hualalai, Hawai`i	*Hibiscadelphus hualalaiensis*
3b	Involucral bracts 18–35 mm long; corolla grayish green externally, dark magenta internally, (5–)6–7 cm long; Mauna Loa, Hawai`i	*Hibiscadelphus giffardianus*
4a	Involucral bracts mostly 4(–5), (20–)23–27(–30) mm long	*Hibiscadelphus crucibracteatus*
4b	Involucral bracts 5–7, 9–22 mm long.	5
5a	Leaf lamina glabrate on both surfaces or with minute, scattered stellate trichomes only on principal veins, the trichomes sparsely tufted in principal vein axils; Kalalau Valley, Kaua`i	*Hibiscadelphus woodii*
5b	Leaf lamina sparsely stellate pubescent adaxially, sparsely to densely stellate pubescent abaxially; Maui, Hawai`i	6
6a	Calyx ca. 1.2 cm long; Kohala Mts., Hawai`i	*Hibiscadelphus bombycinus*
6b	Calyx 2.2–2.5 cm long; Maui	7
7a	Plants mostly sparsely pubescent; bracts linear to ligulate or spathulate, apex obtuse to rounded; capsule ovoid; Auwahi, East Maui	*Hibiscadelphus wilderianus*
7b	Plants mostly densely pubescent; bracts linear-subulate to lanceolate, apex acute to acuminate, capsule globose-cuboid to ovoid; Kaua`ula, West Maui	*Hibiscadelphus stellatus*

## Supplementary Material

XML Treatment for Hibiscadelphus stellatus
